# d-Glucose sensor based on ZnO·V_2_O_5_ NRs by an enzyme-free electrochemical approach†

**DOI:** 10.1039/c9ra06491e

**Published:** 2019-10-07

**Authors:** Mohammed M. Rahman, Mohammad Musarraf Hussain, Abdullah M. Asiri

**Affiliations:** Chemistry Department, Faculty of Science, King Abdulaziz University P.O. Box 80203 Jeddah 21589 Saudi Arabia mmrahman@kau.edu.sa mmrahmanh@gmail.com; Department of Pharmacy, Faculty of Life and Earth Sciences, Jagannath University Dhaka-1100 Bangladesh mmhussain@pharm.jnu.ac.bd m.musarraf.hussain@gmail.com

## Abstract

A simple wet-chemical technique was used to prepare zinc oxide-doped vanadium pentaoxide nanorods (ZnO·V_2_O_5_ NRs) in an alkaline environment. The synthesized ZnO·V_2_O_5_ NRs were characterized using typical methods, including UV-visible spectroscopy (UV-Vis), Fourier transform infrared spectroscopy (FTIR), field emission scanning electron microscopy (FESEM), energy dispersive X-ray spectroscopy (XEDS), X-ray photoelectron spectroscopy (XPS), and X-ray powder diffraction (XRD). The d-glucose (d-GLC) sensor was fabricated with modification of a slight coating of nanorods (NRs) onto a flat glassy carbon electrode (GCE). The analytical performances, such as the sensitivity, limit of quantification (LOQ), limit of detection (LOD), linear dynamic range (LDR), and durability, of the proposed d-GLC sensor were acquired by a dependable current–voltage (*I*–*V*) process. A calibration curve of the GCE/ZnO·V_2_O_5_ NRs/Nf sensor was plotted at +1.0 V over a broad range of d-GLC concentrations (100.0 pM–100.0 mM) and found to be linear (*R*^2^ = 0.6974). The sensitivity (1.27 × 10^−3^ μA μM^−1^ cm^−2^), LOQ (417.5 mM), and LOD (125 250 μM) were calculated from the calibration curve. The LDR (1.0 μM–1000 μM) was derived from the calibration plot and was also found to be linear (*R*^2^ = 0.9492). The preparation of ZnO·V_2_O_5_ NRs by a wet-chemical technique is a good advancement for the expansion of nanomaterial-based sensors to support enzyme-free sensing of biomolecules in healthcare fields. This fabricated GCE/ZnO·V_2_O_5_ NRs/Nf sensor was used for the recognition of d-glucose in real samples (apple juice, human serum, and urine) and returned satisfactory and rational outcomes.

## Abstract 2

A simple wet-chemical technique was used to prepare zinc oxide-doped vanadium pentaoxide nanorods (ZnO·V_2_O_5_ NRs) in an alkaline environment.

## Graphical abstract

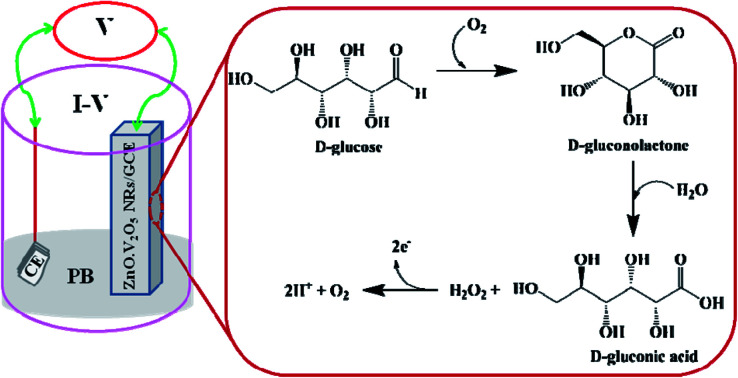

## 1. Introduction

Zinc oxide nanoparticles (ZnO NPs) are a significant *n*-type semi-conductor and have attracted much attention due to their intriguing characteristics, for example, high electron mobility, high transmittance for visible light, and strong luminescence. Several applications, such as acoustic wave devices, light-emitting diodes, piezoelectric transducers, solar cell windows, transparent conductors, sensors, biosensors, gas-sensors, and thin film transistors, of ZnO NPs have been investigated.^1^ A significant amount of research has been devoted to the development of various ZnO nanostructures, such as belts, combs, fibers, particles, ribbons, rods, sheets, spheres, tubes, and wires.^2^ Meanwhile, various oxides of vanadium occur as VO, VO_2_, V_2_O_3_, and V_2_O_5_ depending on the oxidation state of vanadium (V^2+^ to V^5+^). Vanadium pentoxide (V_2_O_5_) can be considered as a promising cathode material. A variety of V_2_O_5_ nanostructures, such as microspheres, nanobelts, nanoflowers, nanosheets, nanowires, and core/shell nanowires, have been reported in earlier studies. V_2_O_5_ possesses excellent chemical and thermal stability, and unique electrochemical and photoelectric characteristics, and is used as a gas sensor, catalyst, window for solar cells, and in electrochromic devices.^3–6^

A sensor is a diagnostic tool in which an element can be combined with a physicochemical transducer for the recognition of a specific constituent. A transducer transforms the indication arising from the interaction of the analyte with the component into a quantifiable signal, such as current or voltage, which can be determined effortlessly.^7^d-Glucose (d-GLC) is a preliminary energy source of the human body, and helps in the formation of metabolic intermediates. However, an inappropriate concentration of d-GLC in blood leads to diabetes mellitus and can cause various complications, such as blindness, cardiac, ocular, and peripheral vascular diseases, stroke, and kidney failure. The human body regulates blood d-GLC levels at a concentration of 70–120 mg dL^−1^. Diabetic patients demonstrate a markedly high d-GLC level because they are unable to control their sugar level.^8–10^ The monitoring of d-GLC in biotechnology, clinical diagnosis, and the food industry is growing in current days accompanied by its stable enhancement using a number of procedures. Generally, electrochemical sensors for d-glucose detection can be classified into two types: enzymatic and non-enzymatic. A lot of research has been performed on non-enzymatic d-glucose sensors (NEDGSs) with the purpose of overcoming the drawback of the mediators and enzymes used in such biosensors. NEDGS represent the 4th generation of d-GLC sensor, and are simpler to organize, inexpensive, and have advanced stability compared with other conventional d-GLC sensors. Usually in biosensors, an enzyme acts as a catalyst, but in NEDGS the character of the modifier is of great significance since atoms at the surface of the electrode act as electrocatalysts. A diversity of materials, such as carbon nanotubes, graphene oxide, hybrids, metal oxides or hydroxides, noble metals, and polymers, have been advanced as NEDGSe for their response to the electrocatalytic oxidation of d-glucose. Different metal oxides and bimetallic oxide composites have been proposed as NEDGSs due to their enhanced synergistic catalytic effect. Two aspects can extensively affect the electrochemical performances of a sensor: the technique used for the preparation of the metal oxide and its interaction with the support electrode, and the characteristics of the support electrode (electron transfer rate, geometry, and surface area).^11^ Different approaches have been developed for the identification of d-glucose, such as: (i) colorimetry, (ii) chromatography, (iii) electrochemistry, (iv) electrochemiluminescence, (v) infrared spectroscopy, (vi) flow injection analysis, (vii) fluorimetry, (viii) Raman spectroscopy, (ix) photo-electrochemistry, (x) quantum dots, (xi) optical polarization rotation measurement, (xii) photo-acoustic probes, (xiii) surface plasmon resonance, (xiv) thermometric, (xv) enzymatic, (xvi) magnetic, (xvii) piezoelectric, (xviii) optical measurement, and (xix) oxygen sensor techniques.^12–17^ However, these methods tend to be expensive, time consuming, and extra care is necessary in handling. Hence, it remains necessary to design an accurate, simple, highly sensitive, less expensive, non-invasive, user-friendly, responsive, quantitative, specific, and real-time monitoring system as a d-GLC sensor, in particular, in order to avoid the micro- and macrovascular complications of other typical detection methods of d-glucose.^18–20^ This research work demonstrated the design, categorization, and development of a d-glucose sensor based on ZnO·V_2_O_5_ NRs and Nafion (Nf) using an *I*–*V* method. The proposed GCE/ZnO·V_2_O_5_ NRs/Nf sensor was tested by applying it to the determination of the d-glucose concentration in spiked biological and food samples, and achieved good results.

## 2. Experimental

### 2.1 Materials and method

Analytical-grade reagents, including EtOH, Nafion (Nf), NaOH, ascorbic acid, l-aspartic acid, cholesterol, d-glucose, GABA, glycine, l-cysteine, l-tyrosine, tannic acid, and uric acid, were purchased from Sigma-Aldrich Company, KSA, and used as received FTIR and UV-Vis spectra of the off-white ZnO·V_2_O_5_ NRs were recorded on a Thermo Scientific NICOLET iS50 FTIR spectrometer and Evolution 300 UV-Vis spectrophotometer, correspondingly. Electrochemical criteria (arrangement, morphology, elemental analysis, and particle size) of the ZnO·V_2_O_5_ NRs were evaluated by means of an FESEM instrument (JEOL, Japan) with XEDS attached. XRD experiments were also performed under ambient conditions to determine the crystalline pattern of the ZnO·V_2_O_5_ NRs. XPS experimentation was conducted for the determination of the binding energies among Zn, V, and O on a K-α_1_ spectrometer (K-α_1_ 1066, Thermo Scientific) with an excitation radiation source (A1 Kα_1_). The *I*–*V* procedure was conducted at a particular point using the modified GCE with ZnO·V_2_O_5_ NRs on a Keithley electrometer (USA, 6517A) in order to determine the biomolecules.

### 2.2 Preparation of the ZnO·V_2_O_5_ NRs

The ZnO·V_2_O_5_ NRs were synthesized by using an easy weight chemical technique (WCT)^21,22^ from the reacting precursors of zinc chloride (ZnCl_2_), vanadium chloride (VCl_3_), and NaOH. WCT is an established solid-state procedure and is extensively applied in the preparation of undoped or doped nanomaterials. In this procedure, ZnCl_2_ (13.79 g) and VCl_3_ (7.86 g) were dissolved in distilled water (DW) in two separate round-bottom flasks for the preparation of mother solutions of ZnCl_2_ (100.0 mM and 1.0 L) and VCl_3_ (100.0 mM and 500.0 mL) under constant stirring. Doping solutions of the NRs of ZnO·V_2_O_5_ (100.0 mL) were made from these mother solutions in 1 : 1, 1 : 2, 1 : 3, and 1 : 4 ratios and the pH of the consequential solutions (Table S1†) was controlled with the addition of NaOH and then the solutions were positioned on a hot plate at 90.0 °C with continuous stirring. The flasks were cleaned with water and acetone accordingly after 6.0 h of continuous stirring and subsequently kept for solvent evaporation in the open air (24 h) at room temperature. Secondary ZnO·V_2_O_5_ NRs were dried in the oven at 60.0 °C (24 h), ground into powders, and again dried at 60.0 °C in the oven (24 h) consecutively for use in the optical characterization and for application. A feasible mechanism for the growth of ZnO·V_2_O_5_ NRs is as follows (eqn i–v).

i ZnCl_2_ + 2NaOH → Zn(OH)_2(aq)_ + 2NaCl_(s)_↓

ii Zn(OH)_2(aq)_ → ZnO_(s)_↓ + H_2_O

iii VCl_3_ + 3NaOH → V(OH)_3(aq)_↓ + 3NaCl_(s)_↓

iv 2V(OH)_3(aq)_ → V_2_O_5(s)_↓ + H_2_O + 2H_2_↑

v ZnO_(s)_ + V_2_O_5(s)_ → ZnO·V_2_O_5(s)_↓

**Scheme 1:**
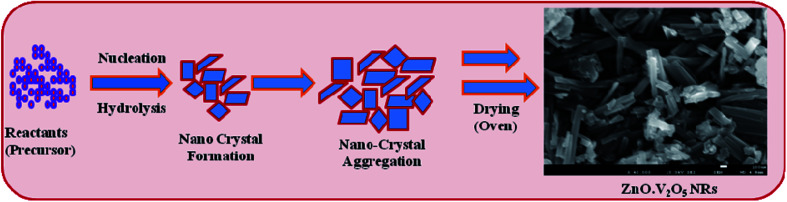
Growth mechanisms of ZnO·V_2_O_5_ NRs.

According to the Ostwald-ripening principle, ZnO·V_2_O_5_ nucleus development is accomplished primarily by common aggregation, followed by re-aggregation of the nanocrystals. Nanocrystals are crystallized and re-aggregated with each other by the counter-part through van der Waals forces and consequently ZnO·V_2_O_5_ NRs morphology was formed in efficiently (Scheme 1). The prepared NRs were examined in detail for their electrochemical properties and then used for the sensing of biomolecules.

### 2.3 Preparation and fabrication of a GCE with ZnO·V_2_O_5_ NRs

A set of phosphate buffers (PB) (pH = 5.7, 6.5, 7.0, 7.5, and 8.0) were prepared from NaH_2_PO_4_ (93.5, 68.5, 39.0, 16.0, and 5.3 mL), Na_2_HPO_4_ (6.5, 31.5, 61.0, 84.0, and 94.7 mL), and DW (500.0 mL). PB (100.0 mM and 10.0 mL) was kept constant throughout the whole exercise. At first, the GCEs were cleaned systematically with water and acetone, and subsequently placed in open air to dry (1.0 h). NRs were dispersed in EtOH to make a slurry and then the slurry was deposited on the dried surface of the GCEs, and kept in open air to dry (1.0 h). A coating binder (Nf) was added drop wise with the deposited NRs and kept again in open air (2 h) till completely dry with uniform development. The customized GCE and Pt wire were used as a working and counter electrode correspondingly with the purpose of illuminating *I*–*V* signals in order to determine the tested biomolecules.

## 3. Result and discussion

### 3.1 Examination of the optical characteristics

Assessing the optical properties offers a considerable distinctiveness for the estimation of the photocatalytic action of the off-white ZnO·V_2_O_5_ NRs. Based on the UV-Vis spectroscopy theory, the band-gap energies of a metal oxide can be acquired because of the adsorption of radiant energy during the shifting of the outer electrons of the atom to a higher energy stage. The UV-Vis spectra of the ZnO NPs, V_2_O_5_ NRs, and ZnO·V_2_O_5_ NRs were recorded at 200–800 nm and expansive absorption bands were found (Table S1†). The theoretical and practical band-gap energies of the prepared ZnO NPs (3.4 and 3.0 eV), V_2_O_5_ NRs (4.8 and 2.9 eV), and ZnO·V_2_O_5_ NRs (1 : 1 to 1 : 4 = 4.5, 4.4, 4.4, and 4.4 eV and 3.0, 2.5, 3.1, and 3.2 eV) were calculated using eqn (vi) and Tauc's equation (vii–ix), respectively (Fig. S1 and S2† and Table S1†).^23–28^vi
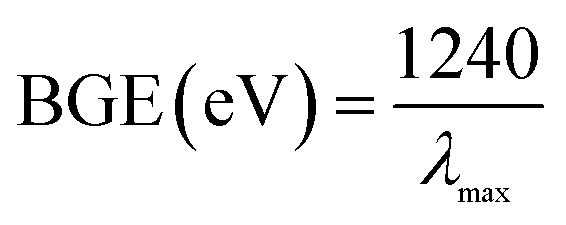

vii *hν* = *A*(*αhν*)^1/*r*^

viii *hν* = *A*(*αhν*)^2^

ix *hν* ∞ (*αhν*)^2^

### 3.2 Evaluation of the structural characteristics

**Fig. 1:**
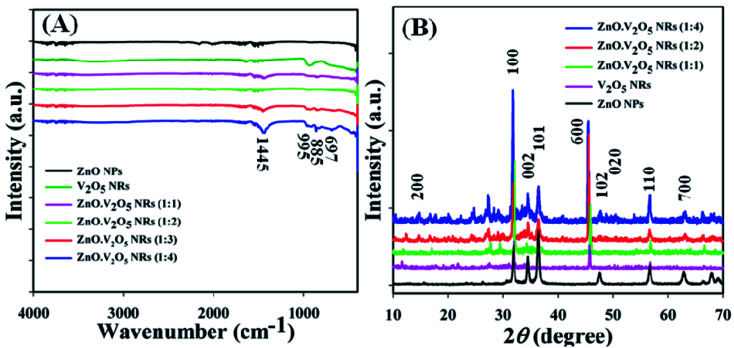
Optical and structural analyses: (A) FTIR and (B) XRD spectra of ZnO NPs, V_2_O_5_ NRs, and ZnO·V_2_O_5_ NRs.

FTIR spectra were recorded (4000–400 cm^−1^) in sequence to identify the functional nature of the ZnO NPs, V_2_O_5_ NRs, and ZnO·V_2_O_5_ NRs under the standard order. The denoted peaks at 1445, 995, 885, and 697 cm^−1^ acknowledged the presence of C–H, Zn–O–V, C–H, and Zn=O in the NRs, correspondingly (Fig. 1A). Two realistic peaks at 995 and 697 cm^−1^ denoted the metal oxide (Zn–O–V) relationship, which recognized the design of the ZnO·V_2_O_5_ NRs.

The crystalline character is the sign of the metal oxide skeleton, and in this regard, XRD investigation was performed (2*θ* range = 10–70°) sequentially to distinguish the crystalline structure of the arranged ZnO NPs, V_2_O_5_ NRs, and ZnO·V_2_O_5_ NRs. A bunch of crystalline peaks with the respective indices based on their strength with the signal at 2*θ* were found as (200), (100), (002), (101), (600), (102), (020), (110) and (700) (Fig. 1B) and all the pragmatic peaks in the spectrum agreed with the JCPDS files 36-1451 and 41-1426. Based on the XRD study, it can be suggested that a significant amount of crystalline ZnO·V_2_O_5_ was presented in the prepared NRs.^3,29,30^ The average particle diameter (*i.e.*, crystallite size) and lattice strains of the NRs were calculated using Scherrer's formula (eqn x), where *D*_p_ = average particle diameter (nm), *λ* = X-ray wavelength (Å), *β* = line broadening (radian), and *θ* = Bragg angle (°).^31^ The average particle diameter was determined for the ZnO NPs (16.80 nm), V_2_O_5_ NRs (86.35 nm), and ZnO·V_2_O_5_ NRs (1 : 1 to 1 : 4 = 47.99, 75.01, 180.10, and 35.96 nm) (Table S2†).x
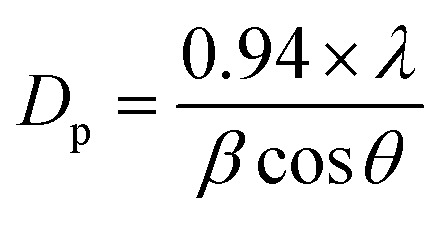


### 3.3 Examination of the morphology and elemental nature

**Fig. 2:**
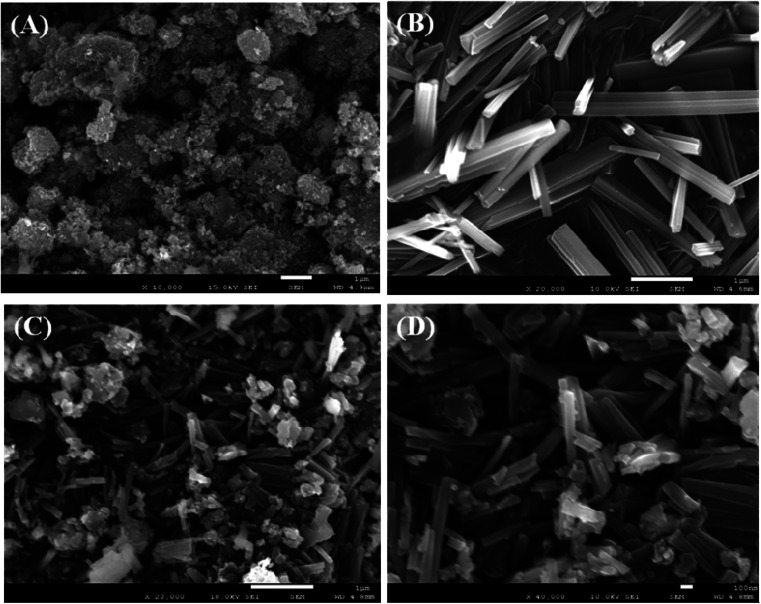
Morphological analysis: (A) ZnO NPs, (B) V_2_O_5_ NRs, and (C and D) ZnO·V_2_O_5_ NRs.

The fundamental nature and morphology of the prepared ZnO NPs, V_2_O_5_ NRs, and off-white ZnO·V_2_O_5_ NRs were examined using FESEM. Distinctive shapes of the ZnO NPs, V_2_O_5_ NRs, and off-white ZnO·V_2_O_5_ NRs were collected at lower to higher magnification (Fig. 2A–D). Depending on the XEDS analysis, zinc (Zn), oxygen (O), and vanadium (V) existed in the off-white ZnO·V_2_O_5_ NRs (Fig. S3A–S3F†). A comparison of the weight % among ZnO NPs, V_2_O_5_ NRs, and ZnO·V_2_O_5_ NRs is presented in Table 1. No auxiliary peaks were found for impurities in the FESEM and XEDS study, which signified that the NRs were composed of Zn, V, and O.

**Table 1:** Elemental analysis, weight (%), and binding energies of the nanomaterials

NM		Weight (%)			Binding energies (eV)				
		O	V	Zn	V^5+^		O 1s	Zn^2+^	
					2p_3/2_	2p_1/2_		2p_3/2_	2p_1/2_
ZnO NPs		42.07	—	57.93	—	—	532.0, 535.4	1022.5	1046.0
V_2_O_5_ NRs		7.59	92.41	—	518.2	524.6	534.7	—	—
ZnO·V_2_O_5_ NRs	1 : 1	51.78	6.05	42.17	517.6	525.8	533.0	1023.0	1046.5
	1 : 2	28.68	9.0	62.31	517.2	522.0	534.6	1026.0	1045.5
	1 : 3	52.30	40.60	7.10	522.0	—	536.0	1026.5	1048.5
	1 : 4	44.95	4.92	50.13	520.0	527.0	534.6	1025.0	1049.0

### 3.4 Examination of the binding energies

**Fig. 3:**
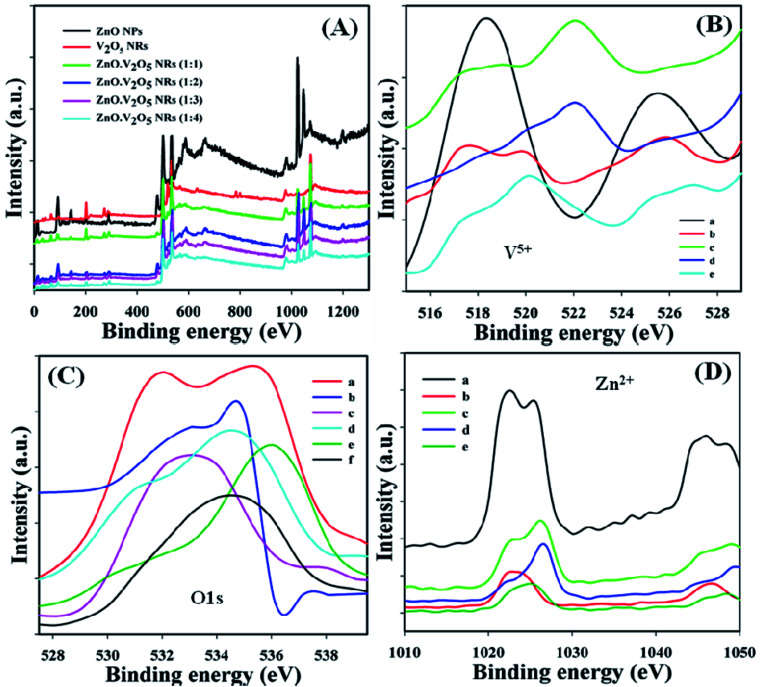
XPS characterization: (A) full spectra, (B): V^5+^ [a: V_2_O_5_ NRs, b–e: ZnO·V_2_O_5_ NRs (1 : 1–1 : 4)], (C) O 1s [a: ZnO NPs, b: V_2_O_5_ NRs, c–f: ZnO·V_2_O_5_ NRs (1 : 1–1 : 4)], and (D): Zn^2+^ [a: ZnO NPs, b–e: ZnO·V_2_O_5_ NRs (1 : 1–1 : 4)].

X-ray photoelectron spectroscopy (XPS) is a quantitative spectroscopic tool and was applied to discover the chemical environment of the fundamental element present in the ZnO·V_2_O_5_ NRs. The kinetic energy and electron number of a nanomaterial may be projected through XPS experiment due to the irradiation of an X-ray with NRs. It was found that zinc, vanadium, and oxygen are present in the synthesized ZnO·V_2_O_5_ NRs from the XPS analysis (Fig. 3A). The spin orbit V^5+^, O 1s, and spin orbit Zn^2+^ spectra were assigned to the major peaks, and the results are presented in Table 1. From these studies, it was recognized that vanadium (V^5+^), oxygen (O^2^), and zinc (Zn^2+^) are present in the NRs (Fig. 3B–D).^3,32–34^

## 4. Application

### 4.1 Determination of d-glucose using ZnO·V_2_O_5_ NRs by the *I*–*V* technique

**Scheme 2:**
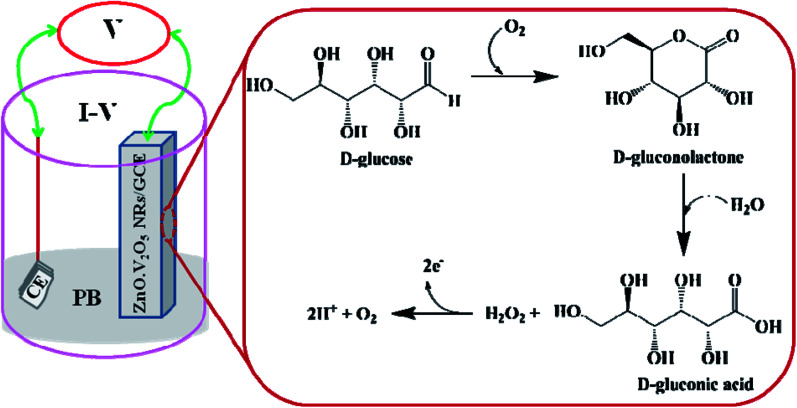
Possible mechanism for the non-enzymatic electro-oxidation of d-glucose to d-gluconic acid in phosphate buffer system.

Extension of the modified electrode with nanorods is the groundwork for utilizing it as a non-enzymatic sensor (NES). A key principle of ZnO·V_2_O_5_ NRs customized as a NES onto the GCE is their point of detection to measure target biomolecules (d-glucose) in the phosphate buffer system. The probable mechanism for revealing d-glucose by the ZnO·V_2_O_5_ NRs using the *I*–*V* performance is presented in Scheme 2, where d-glucose was converted into d-gluconolactone and then d-gluconic acid and hydrogen peroxide upon electro-oxidation. After that, H_2_O_2_ gets converted into oxygen and proton by releasing two electrons.^35,36^ These released electrons are responsible for the current–voltage curve for d-glucose detection.

**Fig. 4:**
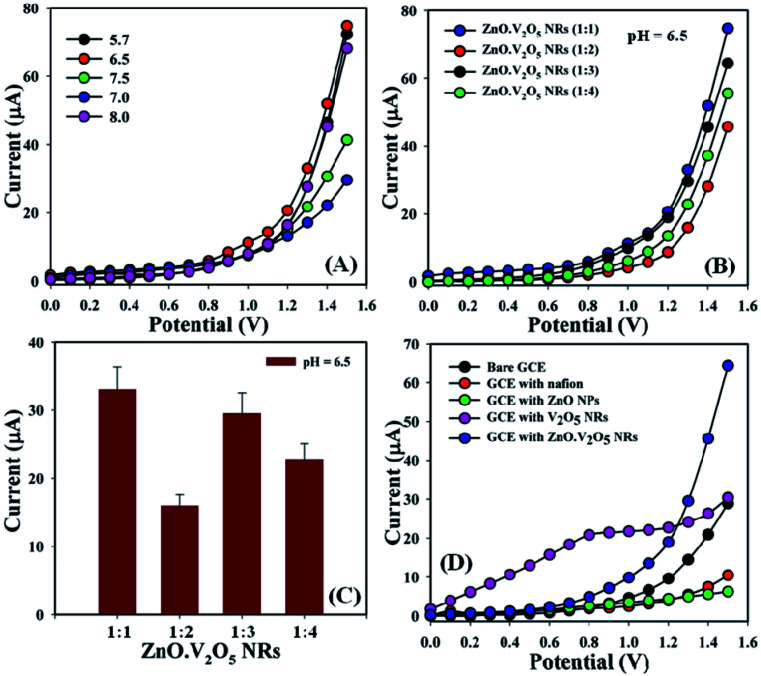
(A) pH optimization, (B) NR examination in terms of pH = 6.5, (C) bar diagram presentation of NRs optimization at +1.3 V with an error limit of 10.0%, and (D) bare and coated GCEs.

A challenging aim of depositing ZnO·V_2_O_5_ NRs onto a GCE as a non-enzymatic sensor was to categorize biomolecules that are not considerably useful in the healthcare field, and here the pH of the diverse phosphate buffer (pH = 5.7, 6.5, 7.0, 7.5, and 8.0) was analyzed to investigate which PB was more appropriate to determine the biomolecules, where pH = 6.5 was found to allow a quick response for *I*–*V* measurements in the entire experiment (Fig. 4A). The ratios of ZnO·V_2_O_5_ NRs were optimized in terms of PB (10.0 mL, pH = 6.5, and 100.0 mM) to detect which ratio was more applicable. In this regard, ZnO·V_2_O_5_ NRs (1 : 1) were found to be more responsive to conduct the selectivity tests for biomolecules (Fig. 4B), where Fig. 4C is the bar-diagram presentation of the ratio optimization at +1.3 V with an error bar, EB = 10.0%. A comparison was performed in phosphate buffer (10.0 mL, pH = 6.5, and 100.0 mM) regarding bare GCE, GCE, with Nf, ZnO NPs, V_2_O_5_ NRs, and ZnO·V_2_O_5_ NRs as the working electrode. A sharp difference in the current responses was found for ZnO·V_2_O_5_ NRs in comparison with the bare GCE, GCE with Nf, ZnO NPs, and V_2_O_5_ NRs (Fig. 4D).

**Fig. 5:**
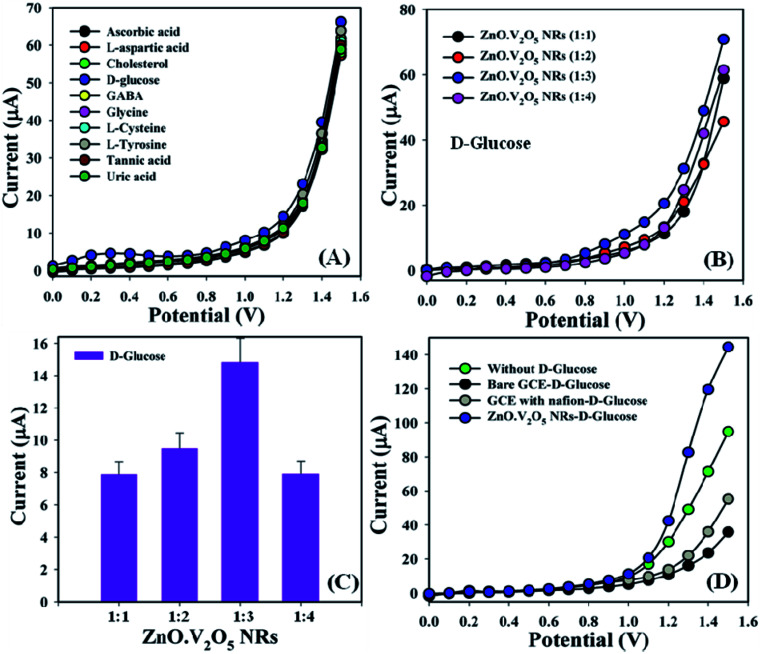
(A) Selectivity study, (B) selectivity optimization, (C) selectivity optimization in the form of a bar diagram at +1.1 V with EB = 10.0%, and (D) in the absence and presence of d-glucose.

Biomolecules, such as ascorbic acid, l-aspartic acid, cholesterol, d-glucose, GABA, glycine, l-cysteine, l-tyrosine, tannic acid, and uric acid (∼25.0 μL and 100.0 nM), were studied in phosphate buffer (10.0 mL, pH = 6.5, and 100.0 mM) using different tailored electrodes in a similar environment to find out the highest current response to the ZnO·V_2_O_5_ NRs (1 : 1), and consequently, it was noticeably observed that the GCE/ZnO·V_2_O_5_ NRs/Nf sensor was more sensitive and selective towards d-glucose compared with other biomolecules (Fig. 5A). Selectivity was also optimized at a d-glucose concentration of 100.0 nM and amount of ∼25.0 μL in terms of the ZnO·V_2_O_5_ NRs ratio, and here ZnO·V_2_O_5_ NRs (1 : 3) appeared to give the major responses toward d-GLC (Fig. 5B), while Fig. 5C gives the bar diagram presentation of the selectivity optimization at +1.1 V with the error bar = 10.0% of ZnO·V_2_O_5_ NRs. The current response without d-GLC, bare GCE-d-GLC, GCE-Nf-d-GLC, and ZnO·V_2_O_5_ NRs-d-GLC were also investigated at ∼25.0 μL and 100.0 nM and it was observed that the GCE/ZnO·V_2_O_5_ NRs/Nf sensor appeared to give the highest response toward d-glucose (Fig. 5D).

**Fig. 6:**
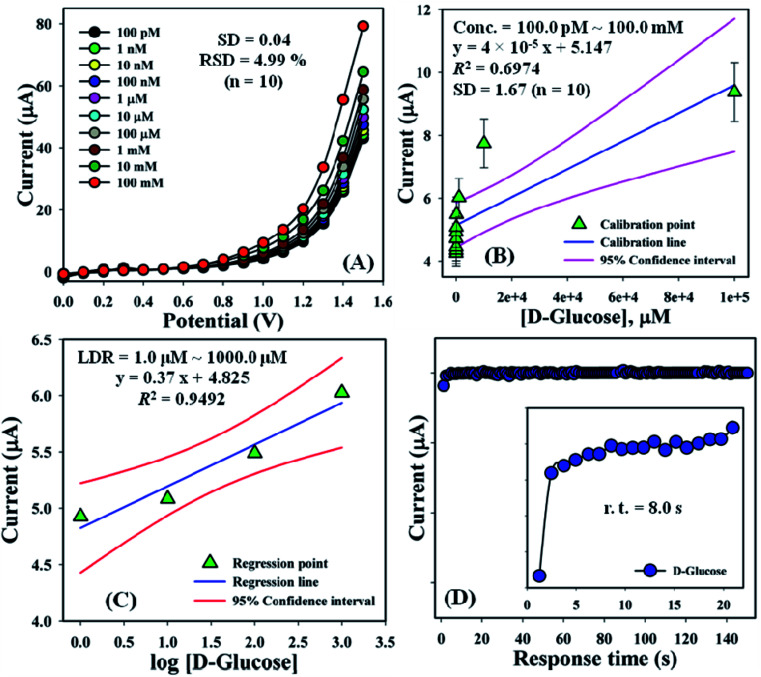
(A) Concentration variation curve of d-glucose, (B) calibration plot at +1.0 V with error bar = 10.0%, (C) LDR curve, and (D) response time of d-glucose toward the GCE/ZnO·V_2_O_5_ NRs/Nf sensor.

An increased current response was reported for the modified GCE/ZnO·V_2_O_5_ NRs/Nf sensor with d-GLC, which offered an enormous exterior region with good exposure in adsorption effectiveness onto the permeable NRs membrane of the marked biomolecule. The *I*–*V* signals of the d-GLC with a range of concentration toward the GCE/ZnO·V_2_O_5_ NRs/Nf sensor were evaluated with indication of the current changes of the customized electrode as a function of the d-GLC concentration in the standard order. It was reported that the current–response augmentation commonly commenced from a lower to higher concentration of d-glucose in a potential range of 0.0 – +1.5 V [SD = 0.04, RSD = 4.99%, and *n* = 10 at +0.5 V] (Fig. 6A). A calibration curve was plotted at +1.0 V with error bar = 10.0% from the d-glucose concentration (100.0 pM–100.0 mM) and was found to be linear (*R*^2^ = 0.6974) (Fig. 6B). The sensitivity (1.27 × 10^−3^ μA μM^−1^ cm^−2^), LOQ (417.5 mM), and LOD (125 250 μM) of the proposed GCE/ZnO·V_2_O_5_ NRs/Nf sensor were calculated using eqn (xi)–(xiii) from the calibration curve, where *m* = slope of the calibration cure (4 × 10^−5^*x* + 5.147), *A* = active surface area of GCE (0.0316 cm^2^), and SD = standard deviation at the calibrated potential (1.67).^37,38^ The LDR (1.0 μM–1000 μM) was derived from commencing the calibration curve and achieved to be also linear (*R*^2^ = 0.9492) (Fig. 6C). The response time of d-glucose (∼25.0 μL and 100.0 nM) toward the GCE/ZnO·V_2_O_5_ NRs/Nf sensor was tested in PB (10.0 mL, pH = 6.5, and 100.0 mM) and found to be 8.0 s (Fig. 6D).xi
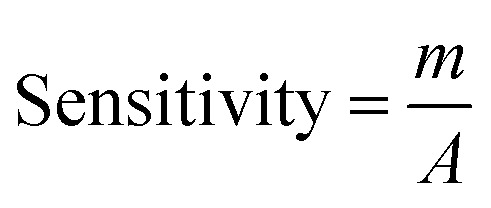
xii
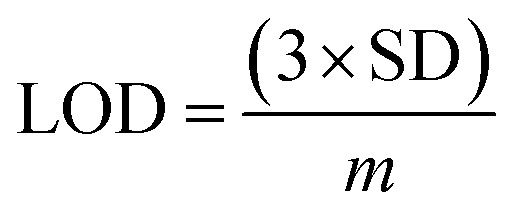
xiii
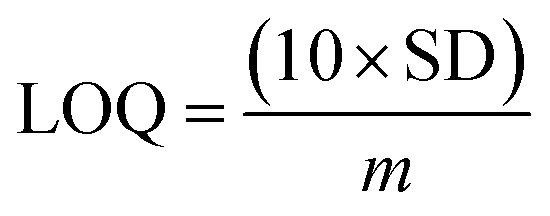


### 4.2 Stability of the sensor

**Fig. 7:**
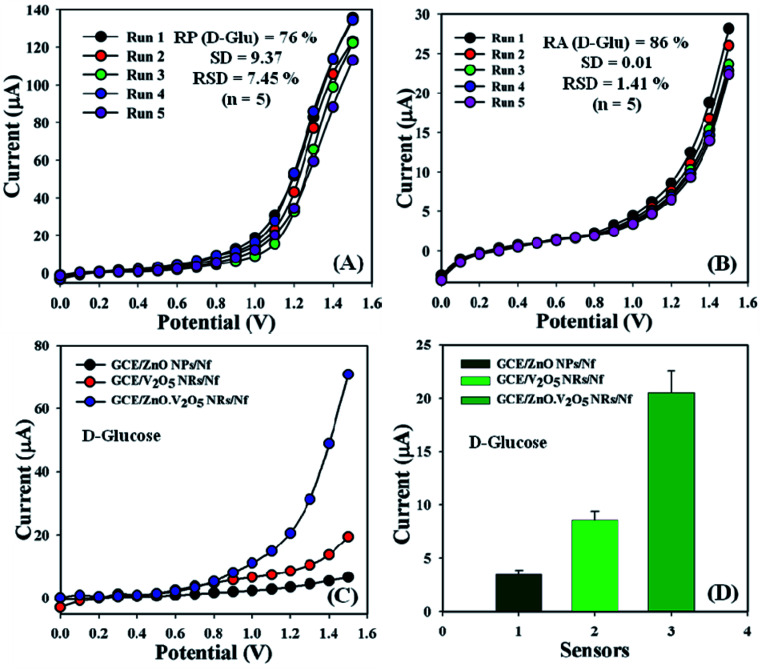
(A) Reproducibility study, (B) repeatability examination, (C) control experiment, and (D) bar diagram presentation of the control experiment at +1.2 V with error bar = 10.0%.

The sensing performance of the GCE/ZnO·V_2_O_5_ NRs/Nf sensor was conducted in PB (10.0 mL, pH = 6.5, and 100.0 mM) using diverse deposited electrodes in a similar environment equal to a few days for the evaluation of the reproducible (RP) aptitude. Accordingly, a series of five successive degrees of d-glucose concentration (100.0 nM) and amount (∼25.0 μL) were tested with the proposed sensor (GCE/ZnO·V_2_O_5_ NRs/Nf) and yielded good RP responses at the calibrated potential (+1.0 V) [RP = 76%, SD = 9.37, RSD = 7.45%, and *n* = 5]. It was accepted that the electrochemical responses did not change broadly after cleaning every experiment for the tailored GCE/ZnO·V_2_O_5_ NRs/Nf sensor (Fig. 7A and Table S1†).

Responses of GCE/ZnO·V_2_O_5_ NRs/Nf sensor were considered significantly with the response time for the purpose of storage capability of proposed sensor at room conditions. The storage ability of the sensor was evaluated using a similar fabricated electrode in PB (10.0 mL, pH = 6.5, and 100.0 mM) and d-glucose concentration (100.0 nM) and amount (∼25.0 μL) under a regular order, and the repeatability (RA) was good at approximately 83.0% of the original value at the calibrated potential (+1.0 V) for the expected sensor (GCE/ZnO·V_2_O_5_ NRs/Nf) for five successive investigations [SD = 0.01, RSD = 1.41%, and *n* = 5] (Fig. 7B and Table S1†). It was markedly reported that the modified GCE/ZnO·V_2_O_5_ NRs/Nf sensor may be used devoid of any considerable changes of the sensitivity over a few days. A control experiment at 100.0 nM was performed in PB (10.0 mL, pH = 6.5, and 100.0 mM), and the GCE/ZnO·V_2_O_5_ NRs/Nf sensor showed a greater response toward d-glucose in comparison with GCE/ZnO NPs/Nf and GCE/V_2_O_5_ NRs/Nf (Fig. 7C), with a bar diagram illustration of the control experiment at +1.2 V with EB = 10.0% presented in Fig. 7D. A comparative analysis of d-glucose identification by using diverse electrodes is presented, wherein our proposed GCE/ZnO·V_2_O_5_ NRs/Nf sensor appeared to have good analytical performance (Table 2).

**Table 2:** Determination of d-glucose using different modified electrodes by electrochemical methods^a^

Electrodes	TOE	MOD	LOD (μM)	LDR (μM)	Sensitivity (μA μM^−1^ cm^−2^)	Ref.
Fe_3_O_4_@SnO_2_/MWNTs	GCE	AM	0.8	1–30000	58.9	39
Au@Ag shell NRs	GCE	AM	0.67	20–7020	33.67	40
CP	GCE	AM	0.33	20–500	0.006	41
ZnO NRs/Au	GCE	AM	0.01	0.1–33	1492	42
PMMA-BSA	GCE	AM	—	200–9100	44.1	43
Nf/Au–Fe_3_O_4_@SnO_2_	ITO-ME	EIS, AM	10	50–1000, 1000–8000	92.14, 15.0	44
Fe_3_O_4_-enzyme-Ppy	MGE	AM	0.3	500–34000	—	45
Fe_3_O_4_-RGO	MGCE	AM	0.15	50–1500	9.04	46
Au-PDA-Fe_3_O_4_	MGCE	AM	6.5	20–1880	—	47
Fe_3_O_4_/Cs/Nf	Pt	AM	6.0	6000–2200	11.54	48
Ferri-COs	SPCE	AM	1.38	∼33300	0.677	49
Fe_3_O_4_@Au/MnO_2_	SPCE	AM	13.2	200–9000	2.52	50
Os-complex	SPCE	AM	30	100–10000	—	51
MnO_2_	SPCE	AM	1.0	11–13900	—	52
ZnO·V_2_O_5_ NRs	GCE	I–V	125 250	1.0–1000	1.27 × 10^−3^	This work

a TOE: Type of electrode, MOD: method of detection, LOD: limit of detection, LDR: linear dynamic range, and AM: amperometry.

### 4.3 Study of the interference effect

**Fig. 8:**
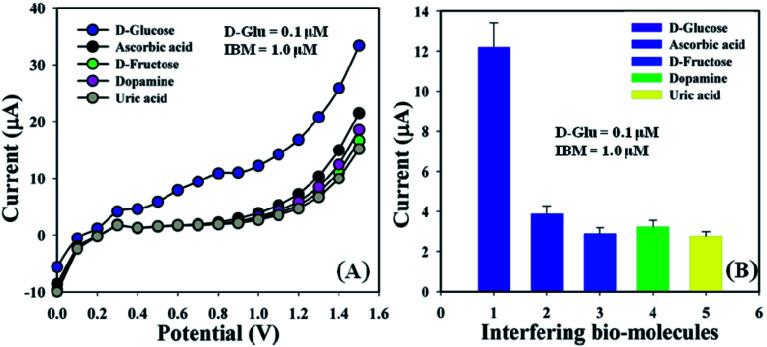
(A) Interference effect of biomolecules and (B) bar diagram presentation of the interference effect at +1.0 V with error bar = 10.0%.

Interference effect examination is one of the most important methods in analytical science, owing to its ability to differentiate the interfering constituents from a biomolecule having a similar physiological background in the biosensor.^53,54^ Ascorbic acid (AA), d-fructose (d-FRCT), dopamine (DP), and uric acid (UA) are generally used as interfering biomolecules (IBM) in non-enzymatic electrochemical d-glucose sensors. The *I*–*V* responses for the GCE/ZnO·V_2_O_5_ NRs/Nf sensor toward the addition of d-glucose (∼25.0 μL and 0.1 μM) and IBM such as AA, D-FRCT, DP, and UA (∼25.0 μL and 1.0 μM) in PB (10.0 mL, pH = 6.5, and 100.0 mM) were recorded. The effects of IBM toward the d-GLC sensor were calculated at the calibrated potential (+1.0 V) and compared with the d-glucose effect, where the interference effect of d-glucose was considered to be 100.0% (Fig. 8A and B and Table 3). It was noticeable that the GCE/ZnO·V_2_O_5_ NRs/Nf sensor did not exhibit any remarkable responses toward the interfering biomolecules. So, the proposed sensor is considered acceptable for the detection of d-glucose with good sensitivity.

**Table 3:** Interference effect studies of biomolecules toward the GCE/ZnO·V_2_O_5_ NRs/Nf sensor^a^

IBM	Observed current				IE (%)	SD (*n* = 3)	RSD % (*n* = 3)
	R1	R2	R3	Average			
d-Glucose	25.24	6.56	4.82	12.20	100	11.32	92.74
Ascorbic acid	4.38	3.72	3.53	3.88	32	0.45	11.51
d-Fructose	3.18	2.80	2.75	2.91	24	0.24	8.08
Dopamine	3.63	3.07	3.03	3.24	27	0.34	10.34
Uric acid	2.91	2.64	2.64	2.73	22	0.16	5.71

a IBM: Interfering biomolecules, R: reading, the interference effect (IE) of d-glucose was considered to be 100%, and SD: standard deviation.

### 4.4. EIS analysis

**Fig. 9:**
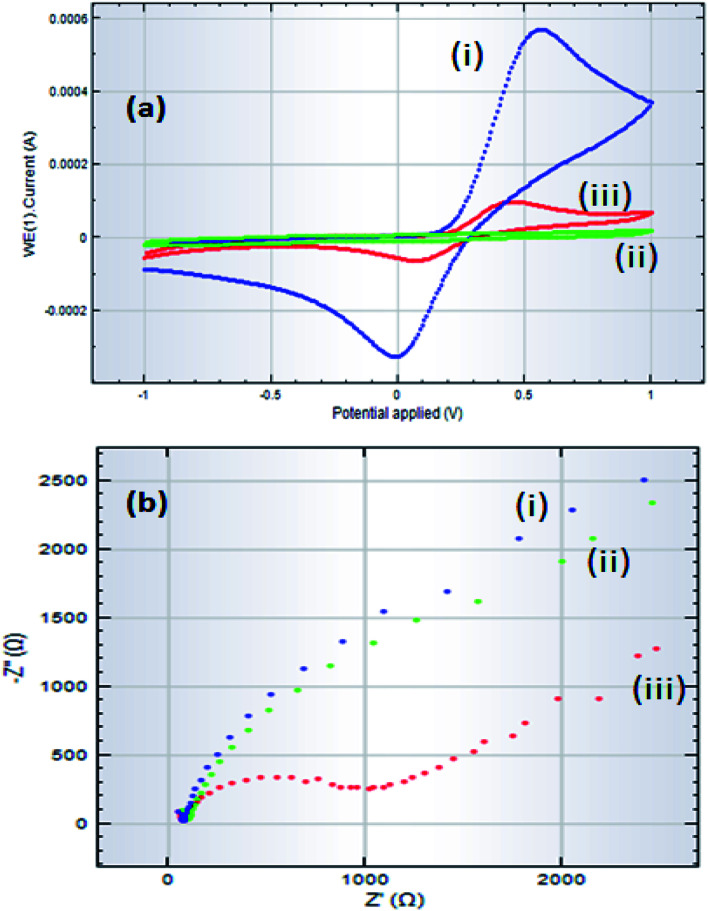
Comparison of the Nyquist plots of bare GCE, GCE/Nf, and GCE/ZnO·V_2_O_5_ NRs/Nf at pH = 7.0 (a) electrochemical voltammetric study and (b) EIS plots of three different modified electrodes recorded with target glucose analyte in 5.0 mM K_3_[Fe(CN)_6_] in 0.1 M PB (pH = 6.5).

Before recommending GCE/ZnO·V_2_O_5_ NRs/Nf as an efficient sensor material for the detection of selective glucose, it was essential to unveil the electrochemical impedance properties of ZnO·V_2_O_5_ NRs in the GCE/ZnO·V_2_O_5_ NRs/Nf assembly. For this purpose, at first, two GCEs were fabricated by immobilizing Nafion and ZnO·V_2_O_5_ NRs compounds, respectively, on two separate GCE surfaces. Next, the impedance (EIS) spectra (5.0 mM K_3_[Fe(CN)_6_] in 0.1 M PB, pH = 6.5) were recorded to explore the relative EIS properties of the electrodes by adjusting to an excitation potential of +0.38 V between 0.1 Hz and 0.1 MHz frequency in the presence of glucose, as shown in Fig. 9. For the electron-transfer ability of the various modified electrodes, the electrochemical response by the GCE/ZnO·V_2_O_5_ NRs/Nf sensor in the presence of the target glucose with 5.0 mM K_3_[Fe(CN)_6_] in 0.1 M PB, pH = 6.5) was measured and the results are presented in Fig. 9a. Here, the voltammetric response was found with respect to bare GCE (i), GCE/Nf (ii), and GCE/ZnO·V_2_O_5_ NRs/Nf and the results are given in Fig. 9a. The complex plane plots displayed in Fig. 9a indicate that in 0.1 M PB/K_3_[Fe(CN)_6_], the GCE/ZnO·V_2_O_5_ NRs/Nf (iii) exhibited a lower capacitive current as well as the lowest impedance compared to the GCE (i) and GCE/Nf (ii) electrodes. The lower capacitive nature of the GCE/ZnO·V_2_O_5_ NRs/Nf sensor suggests that the electron-transfer ability of the GCE/ZnO·V_2_O_5_ NRs/Nf electrode was improved, while the ZnO·V_2_O_5_ NRs were bonded on Nf/GCE. Thus, such a modified electronic property of the GCE suggests that the ZnO·V_2_O_5_ NRs sensor probe perhaps can enhance the electrochemical process. A magnified view of the EIS study is presented in Fig. 9b.

### 4.5. Potential analysis of real samples

**Fig. 10:**
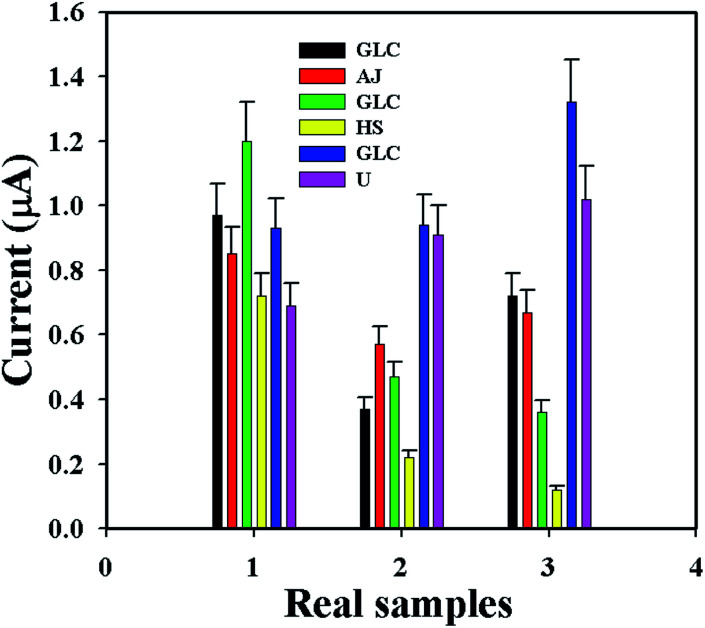
Real sample analysis.

The GCE/ZnO·V_2_O_5_ NRs/Nf sensor was used to determine the d-GLC concentration in different real samples (RSs), such as apple juice (AJ), human serum (HS), and urine (U), by using its *I*–*V* performance. An ordinary adding-up process^55–59^ was applied in the RS investigation to quantify the concentration of d-glucose. A set quantity (∼25.0 μL) of every RS was analyzed in PB (10.0 mL, pH = 6.5, and 100.0 mM) using the GCE/ZnO·V_2_O_5_ NRs/Nf sensor. The found concentrations of d-GLC in the RSs were calculated at +0.3 V with good recovery, which truly recognized that the *I*–*V* design is dependable, suitable, and perfect for the examination of real samples with good results (Fig. 10 and Table 4).

**Table 4:** Analysis of real samples using the GCE/ZnO·V_2_O_5_ NRs/Nf sensor^a^

RS	AC (100 pM → 100 nM → 100 μM)	OAC (μA)		FC	*R* (%)	SD
		Glucose	RS			
AJ	100	0.97	0.85	0.088	88	0.07
	100	0.37	0.57	0.154	154	0.25
	100	0.72	0.67	0.093	93	0.35
	100	1.20	0.72	0.060	60	0.60
HS	100	0.47	0.22	0.047	47	0
	100	0.36	0.12	0.033	33	0.09
	100	0.93	0.69	0.074	74	0.46
U	100	0.94	0.91	0.097	97	0.72
	100	1.32	1.02	0.077	77	0.06

a RS: Real sample, AJ: apple juice, HS: human serum, U: urine, AC: added concentration, OAC: observed average current, FC: found concentration, *R*: recovery, and SD: standard deviation.

## 5. Conclusion

ZnO·V_2_O_5_ NRs were synthesized using an easy wet-chemical technique in basic medium at room temperature and the optical properties of the prepared ZnO·V_2_O_5_ NRs were characterized by means of electrochemical instruments (UV-Vis, FTIR, FESEM, XEDS, EIS, XPS, and XRD). A simple modification procedure was used to fabricate a GCE with ZnO·V_2_O_5_ NRs using Nf. A sensitive d-glucose sensor was developed, effectively based on the tailored GCE/ZnO·V_2_O_5_ NRs/Nf sensor by means of its electrochemical performance. The sensing parameters of the modified d-glucose sensor were found good in terms of the sensitivity, LOQ, LOD, LDR, response time RP, and RA. So, a proficient procedure can be introduced from this new advancement regarding selective, stable, reproducible, and sensitive sensor development in biological fields in a broad scale.

## Authors' contributions

MMH and MMR designed the experiment. MMH performed the experiment and written the whole manuscript. AMA and MMR supervised this research work and corrected the draft manuscript. All authors approved the corrected manuscript in finally to publish in this journal.

## Conflicts of interest

The authors declared that there is no conflict of interest.

## Supplementary Material

RA-009-C9RA06491E-s001
