# Supplementary material for: d-Glucose sensor based on ZnO·V2O5 NRs by an enzyme-free electrochemical approach
Source: RSC Adv. 2019 Oct 7;9(54):31670–82. doi: 10.1039/c9ra06491e (PMC9073342; doi:10.1039/c9ra06491e)
Supplement: RA-009-C9RA06491E-s001 [file RA-009-C9RA06491E-s001.pdf]

Electronic Supplementary Material (ESI) for RSC Advances.
This journal is © The Royal Society of Chemistry 2019

D-Glucose sensor based on ZnO.V_2O_5 NRs by an enzyme free electrochemical approach†

Mohammed M. Rahman [*a], **Mohammad Musarraf Hussain** [*ab], **Abdullah M. Asiri** [a]

[a] *Chemistry Department, Faculty of Science, King Abdulaziz University, Jeddah 21589, P.O. Box 80203, Saudi Arabia*

[b] *Department of Pharmacy, Faculty of Life and Earth Sciences, Jagannath University, Dhaka-1100, Bangladesh*

***Correspondence to,**

M.M. Hussain: mmhussain@pharm.jnu.ac.bd , m.musarraf.hussain@gmail.com

M.M. Rahman: mmrahman@kau.edu.sa , mmrahmanh@gmail.com

Electronic supplementary materials (ESM)

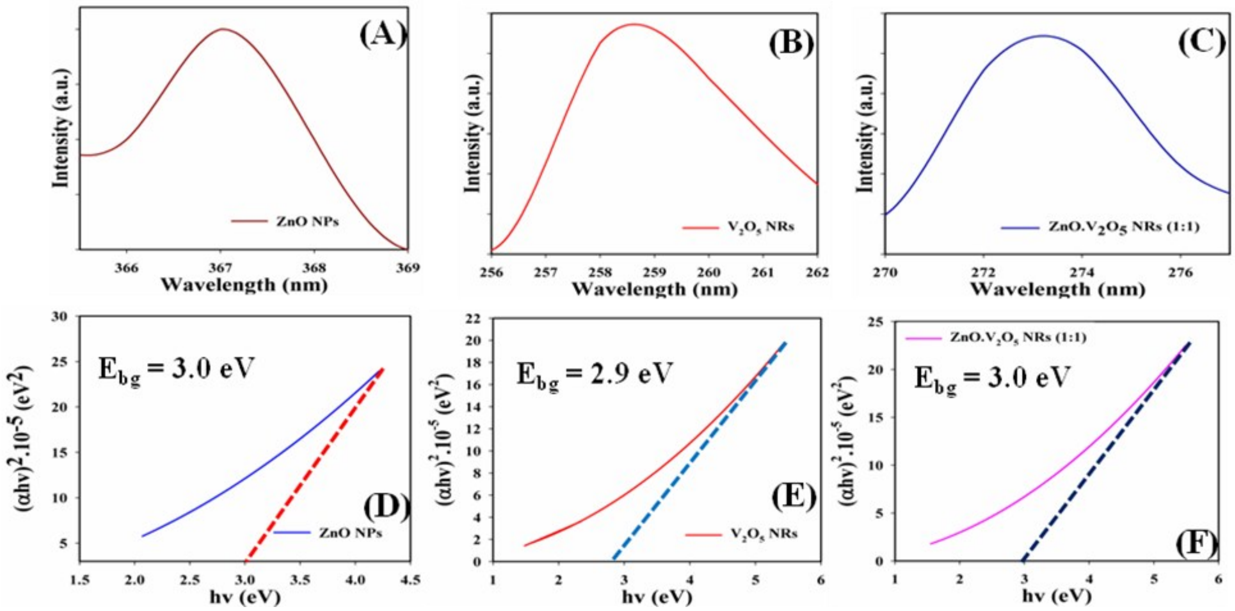

Figure S1: UV spectra and Band gap energy graph, (A-D) ZnO NPs, (B-E) V_2O_5 NRs, and (C-F) ZnO.V_2O_5 NRs (1:1).

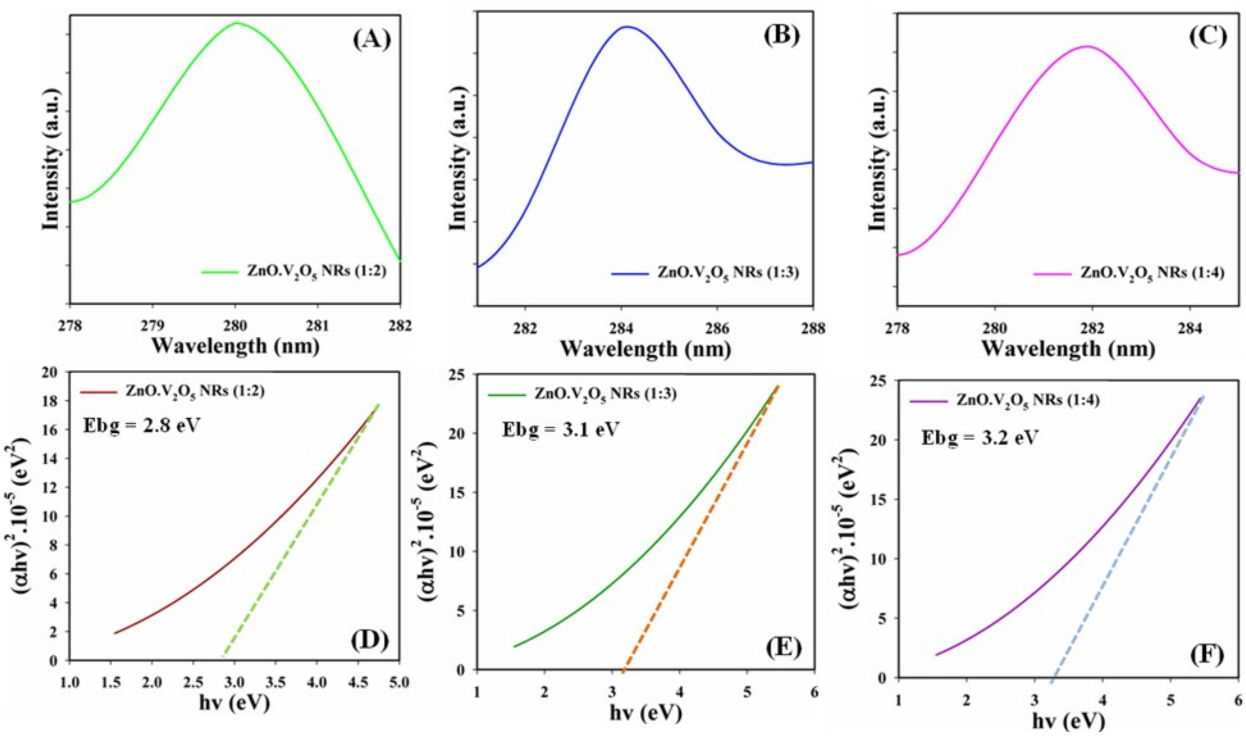

Figure S2: UV spectra and band gap energy plot (A-F) $ZnO.V_2O_5$ NRs (1:2-1:4).

Table S1: BGE of the undoped and doped nanomaterials.

**NMs**		**Physical state**	**pH**	**λ_{max} (nm)**	**Band gap energy (eV)**	
					**Theoretical**	**Practical**
ZnO NPs		White	10.23	367.0	3.4	3.0
V_2O_5 NRs		Deep gray	10.25	258.6	4.8	2.9
	1:1	Light gray	10.27	273.2	4.5	3.0
	1:2	Off white	10.26	280.0	4.4	2.5
$ZnO.V_2O_5$ NRs	1:3	Off white	10.28	284.2	4.4	3.1
	1:4	Deep off white	10.25	281.8	4.4	3.2

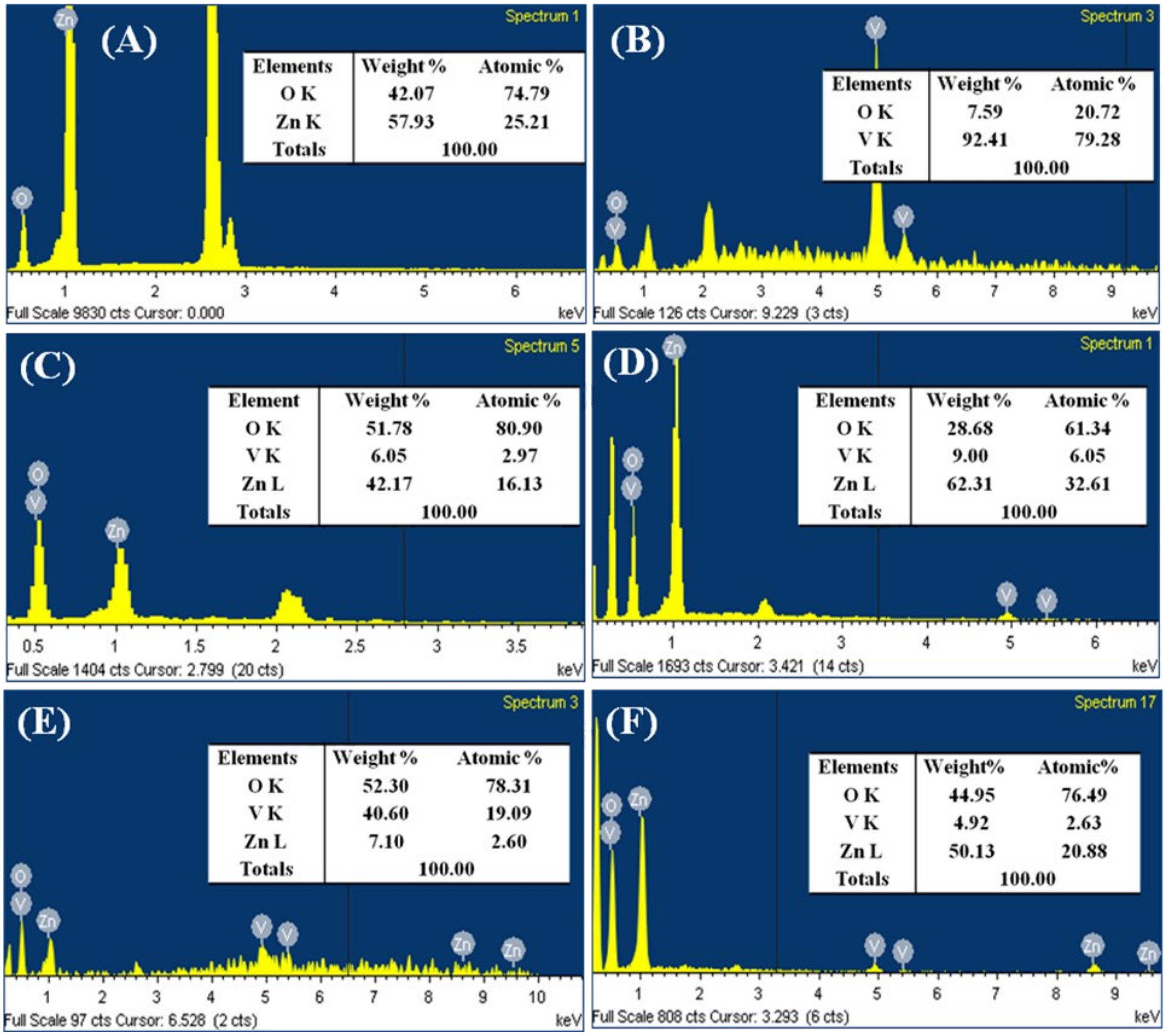

Figure S3: Elemental composition, (A) ZnO NPs, (B) V_2O_5 NRs, and (C-F) ZnO.V_2O_5 NRs (1:1-1:4).

Table S2: Particle diameter and lattice strain of the prepared NM.

NM		***β*** **(º)**	***β*** **(rad)**	**2*θ*** **(º)**	***θ*** **(º)**	***cosθ***	***λ*** **(Å)**	**Dp (nm)**	**LS**
ZnO NPs		0.52	9.07×10^{-3}	36.34	18.17	0.9501		16.80	6.9×10^{-3}
V_2O_5 NRs		0.10	1.74×10^{-3}	31.96	15.98	0.9614		86.35	1.5×10^{-3}
	1:1	0.18	3.14×10^{-3}	32.10	16.05	0.9610	1.54	47.99	2.7×10^{-3}
	1:2	0.12	2.09×10^{-3}	45.50	22.75	0.9222		75.01	1.2×10^{-3}
$ZnO.V_2O_5$ NRs	1:3	0.05	8.72×10^{-4}	45.63	22.82	0.9217		180.10	5.0×10^{-4}
	1:4	0.24	4.19×10^{-3}	31.72	10.86	0.9821		35.96	3.7×10^{-3}

NM = Nanomaterials, β = Line broadening, 2θ = Peak position, θ = Bragg angle, λ = X-ray wavelength, Dp = Average particle diameter, and LS = Lattice strain.

Table S3: Reproducibility and repeatability study of the $ZnO.V_2O_5$ NRs fabricated sensor at calibrated potential (+ 1.0 V).

**Replicates**	**Reproducibility (%)**			**Repeatability (%)**		
	**Current (μA)**	**Individual**	**Average**	**Current (μA)**	**Individual**	**Average**
1	18.90	100		4.43	100	
2	14.46	77		4.40	90	
3	9.14	48	76	3.74	84	86
4	16.36	87		3.58	81	
5	12.32	65		3.40	77	
